# A default mode network subsystem supports both item and associative word encoding: Insights from a meta-analysis

**DOI:** 10.1162/imag_a_00321

**Published:** 2024-10-22

**Authors:** Hongkeun Kim

**Affiliations:** Department of Rehabilitation Psychology, Daegu University, Gyeongsan-si, Republic of Korea

**Keywords:** fMRI, episodic memory, encoding, subsequent memory, default mode network, meta-analysis

## Abstract

Recent meta-analytic evidence has underscored the significant role of the default mode network (DMN) in facilitating item word encoding. This study builds on this finding through a comprehensive meta-analysis of fMRI-based subsequent memory studies that use words as stimuli. The results highlight several key functions within the DMN. Firstly, the dorsomedial prefrontal cortex (dmPFC) subsystem of the DMN plays a pivotal role in enhancing successful word encoding, suggesting its vital involvement in the semantic processing of incoming verbal information. Secondly, the utility of the dmPFC subsystem extends beyond item word encoding to associative word encoding tasks, demonstrating its broad applicability in verbal information encoding. Thirdly, regions within the left inferior frontal cortex, a core component of the dmPFC subsystem, show increased activity during associative compared to item word encoding, emphasizing their role in integrating verbal information with contextual details. Contrary to previous research that linked the DMN with encoding interference—often attributed to the core subsystem’s tendency for mind-wandering—this study highlights the facilitative role of the dmPFC subsystem in memory encoding. The contrasting roles of the DMN subsystems, both interfering and facilitating, challenge traditional views and advocate for a more nuanced understanding of the network’s role in memory encoding.

## 1 Introduction

Understanding the neural basis of episodic memory encoding is a fundamental objective in cognitive neuroscience. Numerous fMRI studies (e.g.,Ranganath et al., 2004;Shrager et al., 2008;Wagner et al., 1998) have employed the subsequent memory paradigm to identify brain regions that are more active during successful versus unsuccessful memory encoding. Meta-analyses of these studies, including those byKim (2011,^2015^), have been pivotal in pinpointing regions consistently involved across diverse experimental setups, providing valuable insights into the neural foundations of successful encoding. However, earlier meta-analyses often encountered challenges in interpretability and applicability, primarily because they amalgamated results from studies with wide variations in experimental parameters such as stimulus type, memory confidence, and the distinctions between item versus associative encoding. These limitations were compounded by the availability of data at the time.

With the increasing number of available studies, more recent meta-analyses (Kim, 2024a,2024b) have adopted stricter inclusion criteria, providing more precise insights that enhance our understanding of the neural mechanisms involved in memory encoding. Notably, a meta-analysis byKim (2024b)focused on identifying key brain regions involved in the successful encoding of words and scenes within item encoding tasks. This analysis revealed a significant role for the default mode network (DMN) in encoding word items, but not scene items, with predominant activity observed in the left inferior frontal cortex, extending to the left middle temporal cortex and left dorsomedial frontal cortex. This observation challenges the conventional perspective that associates increased DMN activity primarily with encoding interference and poorer memory outcomes—often attributed to mind-wandering and engagement in task-unrelated thoughts that detract from task performance (Daselaar et al., 2009;de Chastelaine et al., 2015;Huijbers et al., 2012;Kim et al., 2010;Shrager et al., 2008;Turk-Browne et al., 2006;Wagner & Davachi, 2001).

The contrasting impacts of DMN activation on encoding, which range from positive to negative effects, underscore the complexity of the DMN’s role in memory processes and emphasize the need for more nuanced research. Accordingly, this study delves deeper into the facilitative influence of the DMN on verbal information encoding through an extensive meta-analysis of relevant fMRI studies. It aims to answer three critical questions that further our understanding of how the DMN contributes to memory encoding.

Firstly, the anteromedial prefrontal cortex and medial parietal cortex are traditionally recognized as major hubs of the DMN and have been extensively studied within the broader neuroscience literature (e.g.,Buckner & Carroll, 2007;Qin & Northoff, 2011;Ritchey & Cooper, 2020). However, these components were not highlighted inKim’s (2024b)meta-analysis of word encoding, which instead noted significant findings in the left inferior frontal cortex, middle temporal cortex, and dorsomedial frontal cortex. In an influential study,Andrews-Hanna et al. (2010)categorized the DMN into three subsystems: the core, encompassing the anteromedial prefrontal and posterior cingulate cortex; the dorsomedial prefrontal (dmPFC) subsystem, including the dmPFC, ventrolateral prefrontal cortex, and lateral temporal cortex; and the medial temporal lobe (MTL) subsystem, comprising the retrosplenial and ventromedial prefrontal cortex. Although the topography of word encoding effects in Kim’s meta-analysis suggests a potential alignment with the dmPFC subsystem, the study did not explicitly investigate this aspect. This meta-analysis aims to directly assess the dmPFC subsystem’s involvement in word encoding, responding to this gap in the literature.

Secondly,Kim’s (2024b)meta-analysis specifically focused on item word encoding tasks, where participants study a single word per trial. This study expands this scope to assess whether the DMN involvement also extends to associative word encoding tasks. These tasks require participants to remember both word items and contextual details, such as the position or color of a word, or whether pairs of words were presented together or separately. The DMN’s role in facilitating semantic information processing is well documented (Binder et al., 2009;Irish & Vatansever, 2020;Kim, 2016;Renoult et al., 2019). Numerous studies (Badre & Wagner, 2007;Devlin et al., 2003;Nozari & Thompson-Schill, 2016;Poldrack et al., 1999) have highlighted the critical function of the left inferior frontal cortex, a key component of the DMN’s dmPFC subsystem, in processing verbal information semantically. Given the extensive semantic demands of both item and associative word encoding tasks, it is hypothesized that the DMN’s involvement is crucial across both task types.

Finally, this study investigates whether the DMN is engaged differently during item and associative word encoding. Associative word encoding tasks involve integrating verbal information with contextual details, adding complexities not present in single-word encoding. Although a well-known hypothesis predominantly associates such functions with the hippocampus, the evidence is mixed. Some studies confirm this link (Davachi et al., 2003;Diana et al., 2007;Ranganath et al., 2004;Rugg et al., 2012), while others do not find support (Gold et al., 2006;Kirwan et al., 2008;Shrager et al., 2008). The working hypothesis of this research posits that the DMN’s role in memory encoding extends beyond mere semantic processing to include the integrative encoding of verbal information with its contextual nuances. This suggests a more pronounced engagement of the DMN during associative compared to item word encoding. Shifting away from the traditional hippocampus-centric perspective in associative encoding studies, this research highlights the broader functional contributions and dynamic network interactions of the DMN.

It is important to clarify that significant involvement of the DMN in associative word encoding does not necessarily confirm its role in integrating verbal information with contexts, as this could simply reflect semantic processing of incoming verbal information. However, more pronounced engagement of the DMN in associative compared to item word encoding could provide stronger evidence for its specific role in associative processing beyond mere semantic processing.

## 2 Method

### 2.1 Data collection process

To compile the necessary studies for this meta-analysis, an extensive search was conducted on the PubMed database in December 2023, using a combination of key phrases: “fMRI” with “subsequent memory” or “memory encoding.” To ensure comprehensive coverage, the initial search was supplemented by a Google Scholar search employing similar keywords. Additionally, reference lists of selected articles were manually reviewed. Each identified article underwent a rigorous screening process based on a predefined set of inclusion and exclusion criteria. These criteria and their rationales are detailed in the following sections.

#### 2.1.1 Foundational criteria

To be included in the meta-analysis, studies needed to meet several key criteria. Firstly, they were required to use fMRI as their primary investigative method. Secondly, the participant cohort had to consist exclusively of healthy adults to ensure a homogeneous sample. Thirdly, the studies needed to report data on subsequent memory effects, specifically related to item word encoding, associative word encoding, or both. Lastly, it was essential that the results were presented using standardized brain space coordinates, either Talairach or Montreal Neurological Institute (MNI).

#### 2.1.2 Stimulus types

The meta-analysis was limited to studies using words as stimuli. To further refine the selection, studies involving emotionally charged or reward-associated words (e.g., words linked to monetary gains) were excluded. This decision was informed by findings, such as those in a review byKim (2022), demonstrating that emotional or reward-tied stimuli elicit distinct neural activation patterns compared to neutral stimuli. By focusing solely on studies using neutral words, this meta-analysis aims to ensure a consistent and clear focus, thereby enhancing the reliability and relevance of its findings.

#### 2.1.3 Memory confidence

The initial literature review indicated that item word encoding studies often required participants to rate their confidence during recall, with analyses focusing on high-confidence recollections. However, associative word encoding studies less frequently included such confidence assessments, resulting in fewer analyses of confidently recalled associative information. To minimize potential biases stemming from this discrepancy, the meta-analyses for both item and associative encoding effects included only studies that compared all remembered trials—regardless of confidence level—to those forgotten, deliberately excluding analyses confined to confidently remembered trials. This approach was intended to ensure a more equitable comparison between item and associative encoding studies.

#### 2.1.4 Memory tasks and contrasts

The meta-analysis included studies that focused on either item word encoding or associative word encoding tasks. For item word encoding, studies needed to present a single word per trial and utilize an old/new recognition task during the testing phase. The analyses in these studies concentrated on contrasts between trials where the word was subsequently recognized versus those where it was not.

Associative word encoding studies comprised two types: word-source and word-word associations. In word-source association studies, participants memorized words presented within specific contexts, such as spatial positioning or background color, and were tested on their ability to recall both the words and their associated contexts. The analyses in these studies focused on contrasts between trials where both the word and context were correctly recalled versus those where only the word or neither was recalled correctly. Word-word association studies involved participants studying pairs of words and, during testing, determining whether the pairs were presented as intact, rearranged, or new. The analyses in these studies targeted contrasts between trials where the pairs were correctly identified as intact versus those identified as rearranged or new. This inclusion strategy was designed to accommodate diverse methodologies across studies, ensuring a comprehensive analysis.

Some studies utilizing word-source encoding tasks assessed item word encoding effects by contrasting item-only-correct trials with item-forgotten trials. However, this method risks conflating item memory results with indicators of weaker memory strength, as item-only-correct trials often reflect weaker memory compared to trials where both item and source information are correctly recalled, as supported by memory confidence analyses (Gold et al., 2006;Kirwan et al., 2008). To avoid bias in the assessment of item encoding effects, contrasts involving item-only-correct trials were excluded from this analysis.

#### 2.1.5 Whole-brain analysis

This meta-analysis included only studies that reported activations in whole-brain coordinates, excluding those that limited their analyses to predefined regions of interest (ROI) or employed small volume correction (SVC). This approach was essential because coordinate-based meta-analyses inherently assume that each brain voxel has an equal a priori probability of activation. However, in cases where studies presented findings from both ROI/SVC and whole-brain analyses, activations identified through ROI/SVC methods were included, but only if their*t*or*Z*values exceeded the minimum*t*or*Z*values reported in the whole-brain analysis.

In evaluating the data collection strategy, it is crucial to acknowledge the stringent inclusion criteria employed, which involved exclusively selecting studies that used neutral word stimuli and compared all remembered trials—regardless of confidence level—against forgotten trials. While this restrictive approach may have diminished the statistical power and narrowed the scope of the findings, it was essential for mitigating confounding factors in the comparison of item and associative encoding studies, thereby ensuring the accuracy and consistency of the analysis.

### 2.2 Characteristics of the included studies

This meta-analysis compiled 30 studies that met the inclusion criteria, with further details accessible in the supplementary material online (seeTables S1 and S2, along with Supplementary References). The item word encoding group comprised 20 experiments (where an “experiment” refers to an individual contrast reported in each study) drawn from 18 different studies, with some studies contributing more than one relevant experiment. This group accounted for a total of 134 peak activation foci and included 365 participants. Each experiment in this group compared trials where words were subsequently recognized and those where they were not.

The associative word encoding group incorporated data from 15 experiments derived from 12 different studies, involving a total of 340 participants and 98 peak activation foci. Within this group, 10 experiments were dedicated to word-source associations, with nine employing a comparison format of ‘both word and source-correct greater than word-only-correct.’ An exception was one experiment using ‘both word and source-correct greater than word-only-correct plus forgotten’. The source type was color in three experiments, location in three experiments, task-related contexts in three experiments, and a combination of color and location in one experiment. The remaining five experiments focused on word-word associations. Four of these experiments used the comparison format ‘intact-called-intact greater than intact-called-rearranged’, while one experiment applied ‘intact-called-intact greater than intact-called-rearranged plus forgotten’. In terms of relationships between items, three experiments involved unrelated pairs. One experiment featured a combination of semantically related and unrelated pairs, and the final experiment included a mix of unrelated, semantically related, and phonologically related pairs.

### 2.3 Conducting the meta-analysis

This meta-analysis used the Activation Likelihood Estimation (ALE) algorithm, as detailed byEickhoff et al. (2009,^2011^), utilizing the latest version of GingerALE software (version 3.02), available athttp://www.brainmap.org/ale. The ALE algorithm evaluates the spatial overlap of activation foci across studies to identify areas of consistent brain activity. All activation coordinates reported initially in Talairach coordinates were converted to the Montreal Neurological Institute (MNI) coordinate system using GingerALE’s conversion tool to ensure consistency in the analysis. To ensure data independence, if multiple contrasts from the same participant group were reported within a study, these were combined into a single contrast prior to analysis.

Separate meta-analyses were conducted for both item and associative word encoding effects. Each activation point from the included studies was modeled with a three-dimensional Gaussian distribution to address spatial uncertainties. The full width at half maximum (FWHM) of these distributions was calculated using an advanced ALE approach tailored to the sample size of each study, resulting in median FWHM values of 9.38 mm for item encoding effects and 9.24 mm for associative encoding effects. Cumulative ALE scores for each voxel were calculated by aggregating these Gaussian distributions across all activation points within and across studies. These scores were statistically evaluated against an analytically derived null distribution. Statistical significance was determined using a cluster-level familywise error (FWE) correction set at*p*< 0.05, with a voxel-level threshold of*p*< 0.005 for cluster formation.

A contrast analysis was performed to assess differences between item and associative encoding effects. Studies relevant to both encoding types were aggregated and randomly divided into two groups reflecting their original sample sizes of 18 and 12. Voxel-wise ALE scores were computed for each group, and differences between these scores were assessed. This process was repeated 10,000 times to generate an empirical null distribution for evaluating the statistical significance of the observed differences. Differences in ALE scores were considered significant at a voxel-wise threshold of*p*> 0.95, indicating a 95% probability of a true difference, and required a minimum cluster size of 500 mm^3^to be considered significant.

The resulting thresholded ALE maps were visualized on the three-dimensional, inflated surface model of the Population-Average, Landmark- and Surface-based (PALS) atlas (Van Essen, 2005). To improve the visualization of effects within the hippocampus and other MTL regions, which are less discernible on the cortical surface model of the PALS atlas, additional projections were made onto a specialized brain template developed by the International Consortium for Brain Mapping (Mazziotta et al., 2001).

### 2.4 Associations with intrinsic brain networks

This study investigated the associations between successful encoding effects and intrinsic brain networks by analyzing the distribution of significant voxels, as identified through meta-analyses, within key brain networks. This task necessitated the delineation of major intrinsic networks and their boundaries. The seminal work byYeo et al. (2011)divided the cerebral cortex into seven principal networks: the DMN, frontoparietal network, dorsal attention network, ventral attention network, limbic network, visual network, and somatomotor network. This 7-network model provided the necessary framework for defining network contours.

As outlined earlier in this paper,Andrews-Hanna et al. (2010)categorized the DMN into three primary subsystems: the core, the dmPFC, and the MTL. The distribution of significant voxels within these DMN subsystems was analyzed to understand their roles in successful encoding.Yeo et al. (2011)enhanced their original 7-network model by dividing the cerebral cortex into 17 distinct networks, offering a more detailed granularity. Subsequent research, including those byAndrews-Hanna et al. (2014)andSmallwood et al. (2021), has confirmed that three of these 17 networks closely align with the DMN subsystems initially defined byAndrews-Hanna et al. (2010). This study utilized these specific three networks from the 17-network model to delineate the boundaries of the DMN subsystems.

## 3 Results

### 3.1 Meta-analysis of item and associative word encoding effects

Distinct meta-analyses were conducted to explore subsequent memory effects for item and associative word encoding, followed by a comparative analysis of these effects. Detailed findings are presented inTable 1and illustrated inFigure 1. Notably, both effects were exclusively observed in the left hemisphere, likely reflecting the left hemisphere’s specialization for verbal processing and encoding.

**Table 1.** Summary of the meta-analyses: Subsequent memory (SM) effects for item word encoding, associative word encoding, and comparative analysis.

	Peaks (MNI)			Z	Region	Brodmann area
Volume (mm ^3^ )	*x*	*y*	*z*			
Item SM						
3048	-44	28	-8	5.43	Left inferior frontal cortex	47/46
1920	-44	16	30	4.39	Left middle frontal cortex	9
1704	-58	-42	-8	4.24	Left middle temporal gyrus	20
Associative SM						
5000	-48	28	10	5.04	Left inferior frontal cortex	47/45
1032	-46	-66	28	4.58	Left angular gyrus	39
Associative SM > item SM						
1144	-54	27	8	2.71	Left inferior frontal cortex	45
1024	-30	28	-23	2.66	Left inferior frontal cortex	47
640	-47	-70	20	2.30	Left angular gyrus	39
Item SM > associative SM								
(none)								

**Fig. 1.**
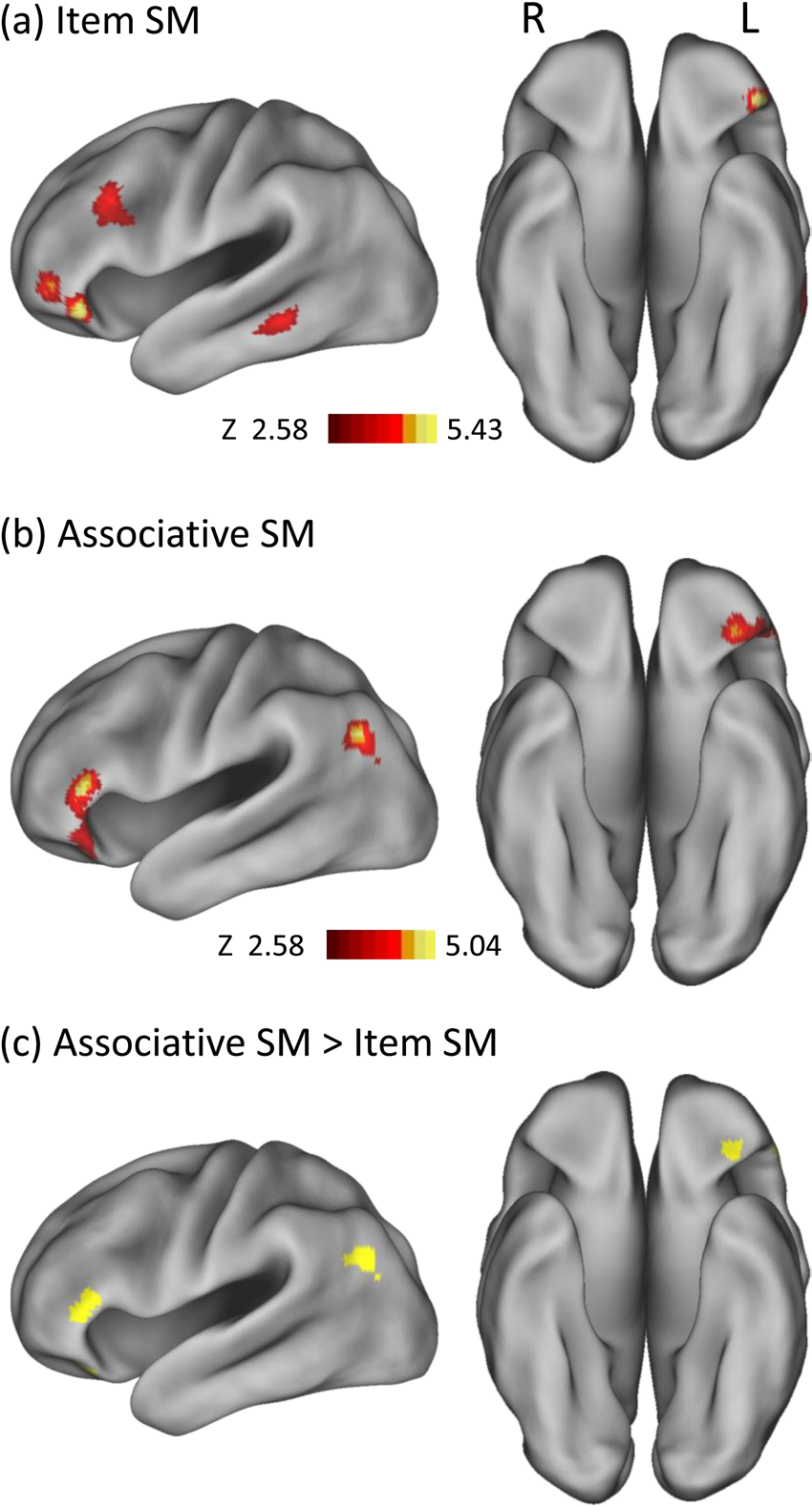
This figure illustrates the significant regions identified by the ALE meta-analysis of subsequent memory (SM) effects in (a) item word encoding, (b) associative word encoding, and (c) comparative analysis. Abbreviations: L = Left hemisphere; R = Right hemisphere.

For item word encoding, significant convergence clusters were identified in the left inferior frontal gyrus (Brodmann areas [BAs] 47/46), the left middle frontal gyrus (BA 9), and the left middle temporal gyrus (BA 20), as shown inFigure 1a. Conversely, for associative word encoding, significant clusters were located in the left inferior frontal gyrus (BAs 47/45) and the left angular gyrus (BA 39), detailed inFigure 1b.

The comparative analysis of the two effects highlighted that specific areas within the left inferior frontal gyrus and the left angular gyrus showed stronger associations with associative effects than with item effects, depicted inFigure 1c. No regions demonstrated stronger associations with item effects compared to associative effects.

### 3.2 Distribution of encoding-related voxels within brain networks

An analysis was conducted to examine the distribution of significant voxels identified in the meta-analyses within seven critical networks, with the results depicted inFigure 2. For item word encoding, the significant voxels were almost evenly distributed between the DMN and the frontoparietal network, accounting for 46.4% and 50.2%, respectively. In contrast, for associative word encoding, the significant voxels were primarily localized within the DMN at 77.8%, with the frontoparietal network contributing only 13.7%. The comparative analysis, highlighting stronger associations with associative effects compared to item effects, revealed that the significant voxels were primarily within the DMN, constituting 74.0% of the effects.

**Fig. 2.**
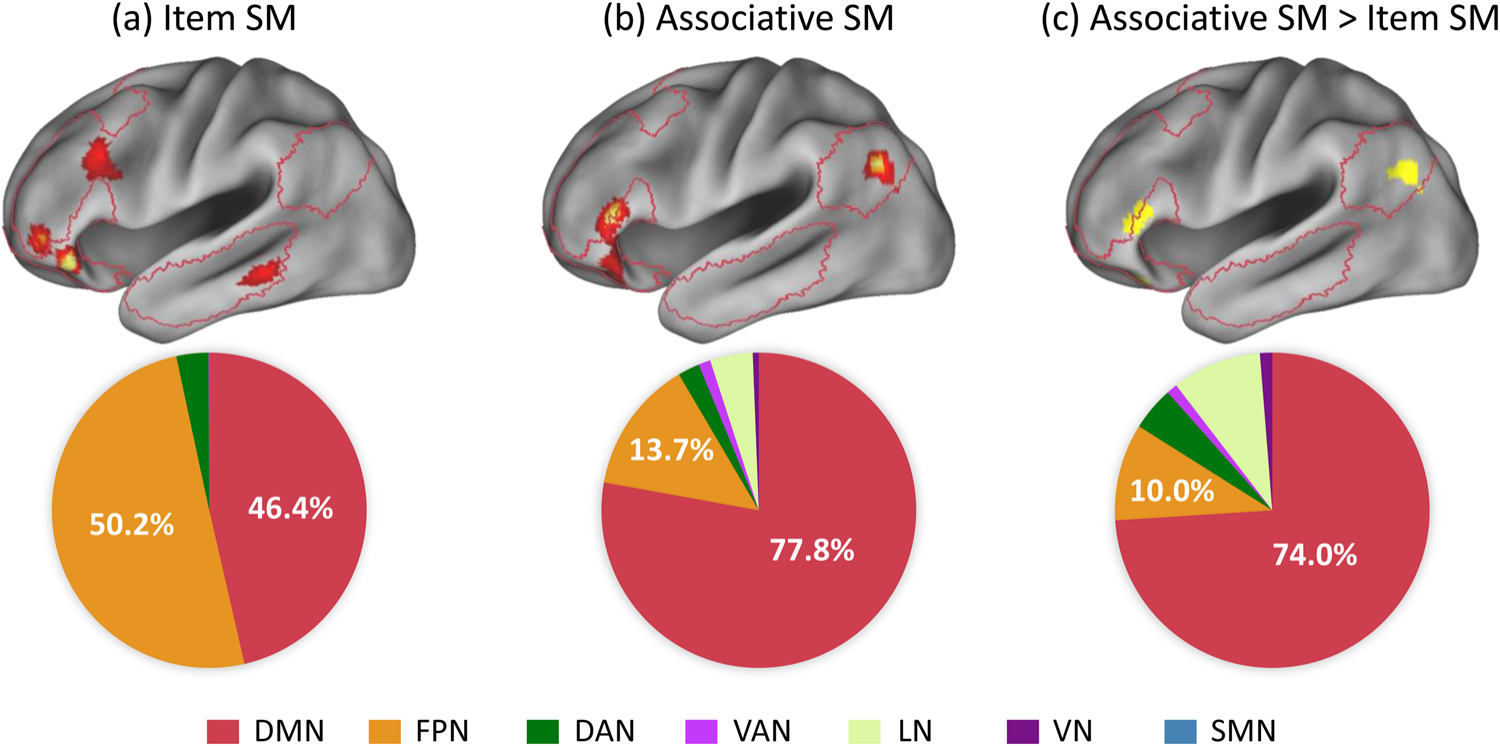
This figure depicts the distribution of significant voxels within Yeo’s 7-network model for the ALE meta-analysis of subsequent memory (SM) effects in (a) item word encoding, (b) associative word encoding, and (c) comparative analysis. Each panel includes a lower section that details the voxel distribution within various intrinsic networks. The percentages are calculated specifically for each effect, enabling direct numerical comparisons between networks within the same effect. The upper section of each panel visually highlights the significant voxels, with crimson lines outlining the estimated boundaries of the DMN, offering a clear visual reference. Abbreviations: DAN = dorsal attention network; DMN = default mode network; FPN = frontoparietal network; LN = limbic network; SMN = somatomotor network; VAN = ventral attention network; VN = visual network.

Further exploration revealed that the distribution of significant voxels within the DMN’s three subsystems was predominantly associated with the dmPFC subsystem across all analyses, as illustrated inFigure 3. Specifically, for item word encoding, the dmPFC subsystem encompassed 100% of the significant voxels within the DMN. For associative word encoding, 79.1% of the significant voxels within the DMN were located in the dmPFC subsystem. The comparative analysis, emphasizing stronger associations with associative effects over item effects, included 71.5% of the significant voxels within the DMN in the dmPFC subsystem.

**Fig. 3.**
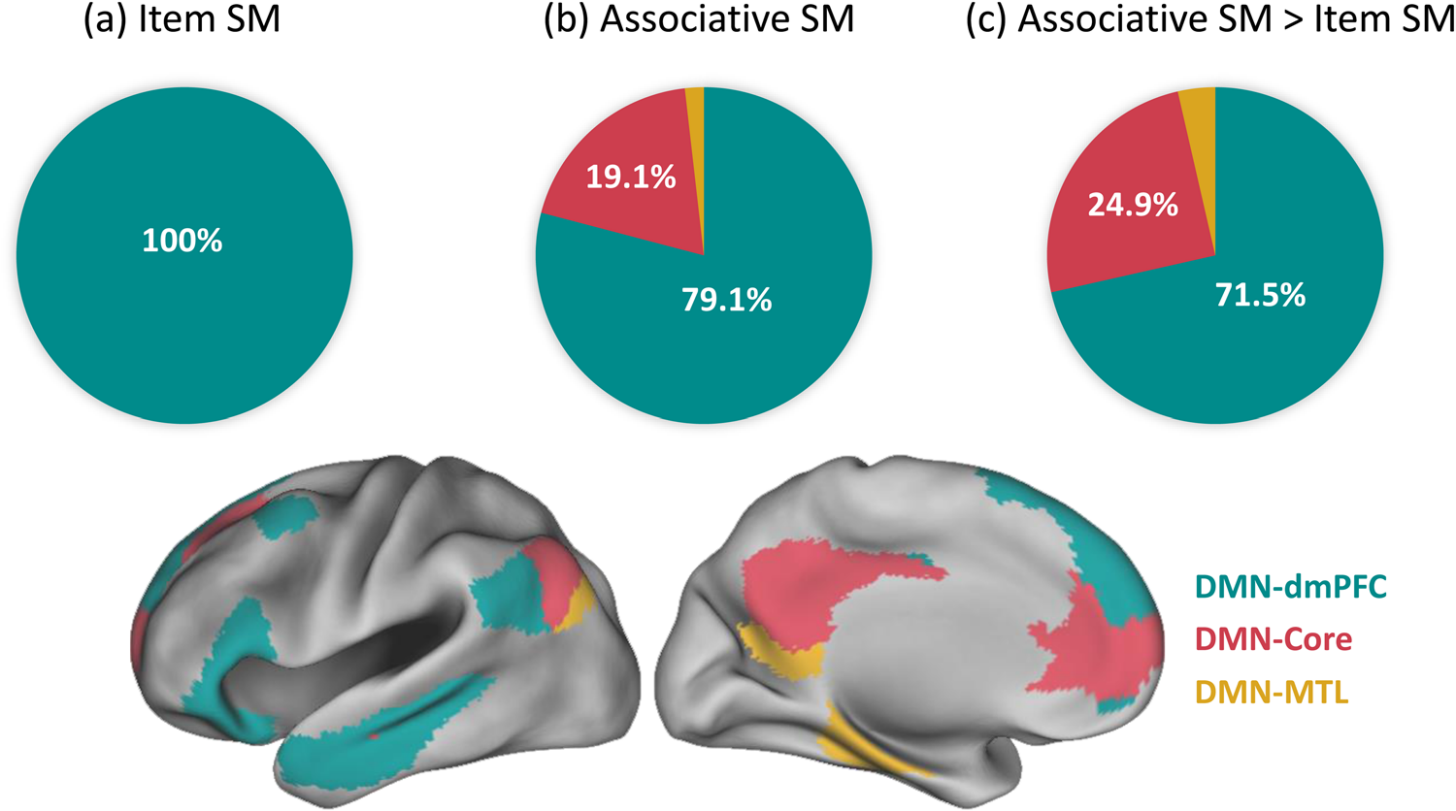
This figure shows the distribution of significant voxels within the three subsystems of the DMN for the ALE meta-analysis of subsequent memory (SM) effects in (a) item word encoding, (b) associative word encoding, and (c) comparative analysis. The delineation of these subsystems corresponds to specific networks identified in Yeo’s 17-network model. These networks are visually depicted in the lower section of the figure, providing a clear reference for the spatial arrangement of each subsystem. The percentages are calculated considering only the voxels within these networks. Abbreviations: DMN = Default Mode Network; dmPFC = Dorsomedial Prefrontal Cortex; MTL = Medial Temporal Lobe.

### 3.3 Supplementary MTL analyses

No significant clusters were initially identified within the hippocampus or adjacent MTL areas for either item or associative word encoding. Given the well-documented role of the MTL in encoding functions, a supplementary analysis was conducted with an adjusted threshold: an uncorrected voxel-level significance of*p*< 0.005 and a minimum cluster size of 200 mm^3^. For item word encoding, this analysis revealed significant engagement in the left hippocampus and parahippocampal cortex (*xyz*= -40, -30, -16), as depicted inFigure 4a. For associative word encoding, significant engagement was observed in the left amygdala and rhinal cortex (*xyz*= -20, -12, -16) and the right parahippocampal cortex (*xyz*= 28, -34, -12), as illustrated inFigure 4b. However, when item versus associative word encoding effects were compared, using a voxel-wise significance threshold of*p*> 0.95 and a minimum cluster size of 200 mm^3^, no significant differences were identified within the MTL regions.

**Fig. 4.**
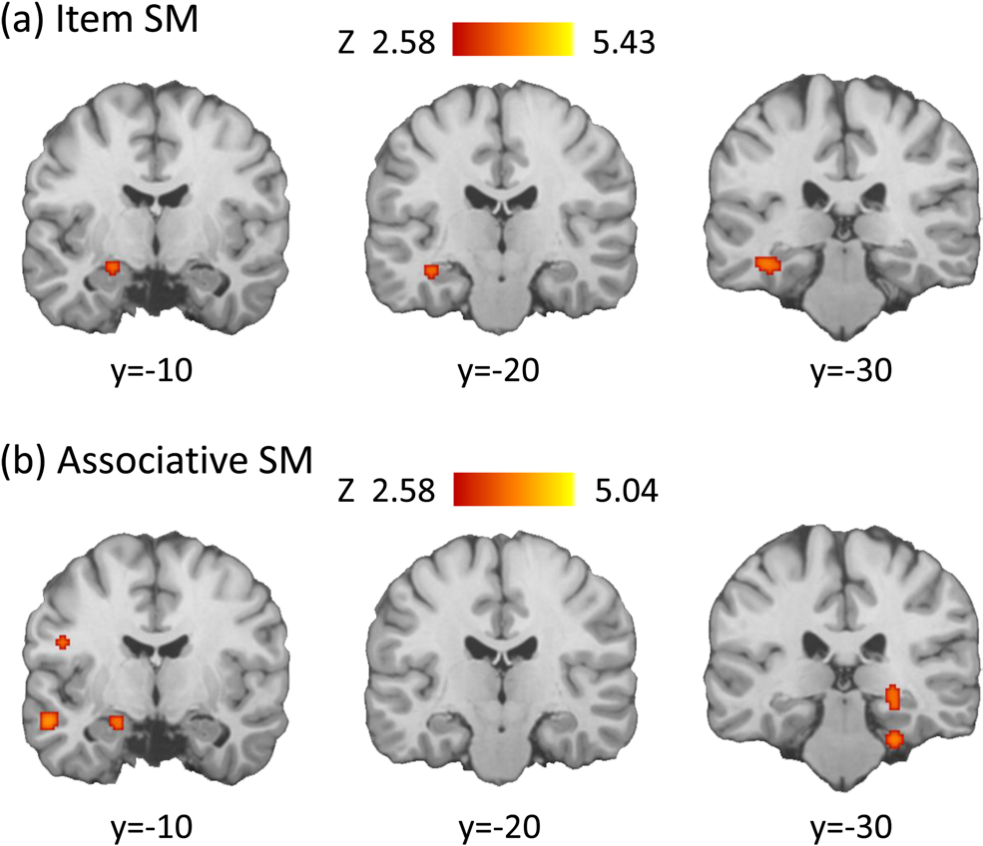
**T**his figure depicts the significant regions within the medial temporal lobe identified by the ALE meta-analysis of subsequent memory (SM) effects for (a) item word encoding and (b) associative word encoding. These findings were obtained from the supplementary analyses that employed lower statistical thresholds—specifically, an uncorrected voxel-level significance of*p*< 0.005 and a minimum cluster size of 200 mm^3^—compared to those used in the main analyses.

**Fig. 5.**
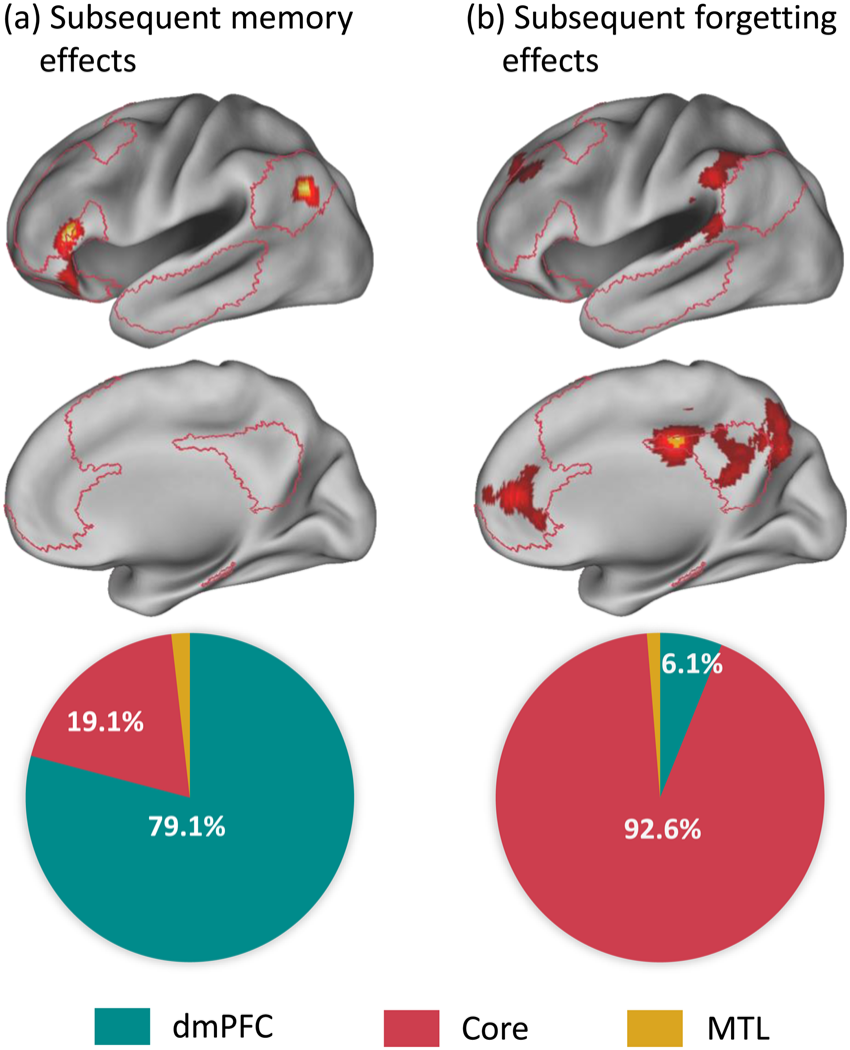
This illustration presents findings from two distinct meta-analyses. Panel (a) displays the results from the current study’s meta-analysis of subsequent memory effects (remembered vs. forgotten) for associative word encoding, incorporating data from 12 studies. Panel (b) shows the results fromKim’s (2018)meta-analysis of subsequent forgetting effects (forgotten vs. remembered), which synthesized data from 53 studies. Each panel includes an upper section that displays significant brain areas, with crimson lines depicting the estimated boundaries of the DMN. The lower section details the distribution of significant voxels within the DMN’s three subsystems: the dorsomedial prefrontal cortex (dmPFC), the core, and the medial temporal lobe (MTL) subsystems. The percentages are calculated based solely on the voxels within these subsystems. The more pronounced findings observed in panel (b) compared to panel (a) may be attributed to the increased statistical power from the larger dataset in the analysis of subsequent forgetting effects. For further explanations, see text.

### 3.4 Impact of excluding studies with item-forgotten trials

Among the 12 studies categorized under associative word encoding, two included item-forgotten trials in the reference condition. To address potential biases from these trials, a supplementary analysis was performed excluding these two studies, applying the same threshold as the main analysis. The main analysis linked associative word encoding to specific regions in the left inferior frontal gyrus and the left angular gyrus. However, the supplementary analysis confirmed this pattern only in the left inferior frontal gyrus (*xyz*= -34, 26, -14), but not in the left angular gyrus. Furthermore, a comparison between item and associative encoding effects revealed a stronger association with associative encoding compared to item encoding solely in the left inferior frontal gyrus (*xyz*= -29, 29, -23), but not in the left angular gyrus. These results highlight a more consistent involvement of the left inferior frontal gyrus in associative word encoding than the left angular gyrus.

### 3.5 Types of associations in convergence clusters of associative encoding

The ALE software includes an analytical tool that identifies specific studies contributing to significant convergence clusters, distinguishing between those reporting significant peak coordinates within these areas and those that did not. This analysis, applied to the significant clusters in associative word encoding, revealed a diverse mix of association types influencing the observed effects. Specifically, the convergence cluster in the left inferior frontal gyrus was supported by seven studies: three focusing on word-word associations, two on word-task associations, one on word-color associations, and one combining word-color and location associations. In contrast, the convergence cluster in the left angular gyrus was derived from three studies: two involving word-task associations and one focused on word-word associations.

### 3.6 Comparing task difficulty in item and associative word encoding

Associative encoding tasks are typically more challenging than item encoding tasks as they require the encoding of both the item and contextual information. This complexity could act as a confounding factor when comparing the neural differentiation between item and associative memory encoding. To address this issue, the corrected recognition rate—calculated as the hit rate minus the false alarm rate—was compared across the two encoding types. The hit rate was defined as the proportion of trials where items were correctly remembered for item encoding, and both item and context were correctly remembered for associative encoding. This metric was available in 11 out of 18 studies for item encoding and 9 out of 12 studies for associative encoding. Analysis revealed that the mean corrected recognition rate was 0.455 (SD = 0.135) for item encoding and 0.410 (SD = 0.144) for associative encoding, which did not differ significantly (*t*(18) = 0.710,*p*= 0.487). However, differences were observed in the mean hit rates—0.641 (SD = 0.123) for item encoding versus 0.532 (SD = 0.102) for associative encoding—resulting in a significant difference (*t*(18) = 2.11,*p*= 0.049). Conversely, the mean false alarm rates—0.186 (SD = 0.082) for item encoding and 0.122 (SD = 0.086) for associative encoding—showed no significant difference (*t*(18) = 1.69,*p*= 0.107). These findings and their implications will be explored further in theSection 4.

## 4 Discussion

### 4.1 Both item and associative word encoding involve the dmPFC subsystem prominently

The meta-analysis revealed that successful item word encoding strongly engaged specific regions in the left inferior frontal gyrus, left middle frontal gyrus, and left middle temporal gyrus. Conversely, successful associative word encoding was predominantly associated with specific areas in the left inferior frontal gyrus and left angular gyrus. Network association analysis indicated that significant findings in both types of encoding were strongly linked with the DMN, accounting for 46.4% of the significant voxels for item word encoding and 77.8% for associative word encoding. Within the DMN, the dmPFC subsystem was predominantly involved, accounting for 100% of the significant voxels in item word encoding and 79.1% in associative word encoding, underscoring its critical importance. These findings suggest that the DMN, especially its dmPFC subsystem, supports both item and associative word encoding.

What are the specific roles of the dmPFC subsystem in word encoding? This study suggests that the dmPFC subsystem plays a pivotal role in the semantic processing of incoming verbal information, a hypothesis supported by previous meta-analytic evidence. It is well established that memory retention improves when information is processed semantically, such as by judging whether a word is concrete or abstract, rather than structurally, such as by judging whether a word is written in uppercase or lowercase (e.g.,Craik & Tulving, 1975;Fletcher et al., 2003;Vidal-Piñeiro et al., 2014). A meta-analysis conducted byKim (2022)linked these depth-of-processing effects primarily to the left inferior frontal cortex—a key component of the dmPFC subsystem. Another meta-analysis (Kim, 2024b) demonstrated that similar regions of the dmPFC subsystem are involved in successful word encoding but not in scene encoding, highlighting their role in semantic and conceptual processing, as opposed to complex perceptual processing.

In contrast to the findings of this study, previous research has linked increased DMN activity to encoding interference and greater forgetting (Daselaar et al., 2009;de Chastelaine et al., 2015;Huijbers et al., 2012;Kim et al., 2010;Shrager et al., 2008;Turk-Browne et al., 2006;Wagner & Davachi, 2001). These effects are often attributed to the network’s roles in mind-wandering and engagement in task-unrelated thoughts, which divert processing resources away from task performance. However, these findings do not contradict the current observations, as they involve a different subsystem within the DMN. The facilitative effects on memory encoding identified in this study are associated with the dmPFC subsystem, while the detrimental impacts observed in previous studies involve the DMN’s core subsystem, as illustrated inFigure 5. This distinction highlights the importance of a nuanced understanding of the DMN’s role in encoding, as previously discussed byMaillet and Rajah (2014).

To differentiate the semantic processes supported by the two subsystems, the dmPFC subsystem is actively involved in encoding external verbal information, leveraging existing semantic knowledge to organize and interpret this data. Conversely, the core subsystem is posited to provide the necessary semantic content and context for internally generated thoughts. This hypothesis posits that the dmPFC and core subsystems act as semantic frameworks or scaffolds for understanding external verbal information and constructing internal thoughts, respectively (for a detailed discussion on semantic scaffolding, seeIrish & Piguet, 2013). Future research is needed to validate this perspective, but it aligns with the observed facilitating and interfering effects of the two subsystems on encoding.

### 4.2 Enhanced engagement of the dmPFC subsystem in associative compared to item word encoding

The direct comparison between item and associative word encoding revealed stronger engagement in specific areas of the left inferior frontal cortex and left angular gyrus during associative encoding compared to item encoding. These areas showed substantial connectivity with DMN, with 74.0% of the significant voxels located within this network. Further analysis highlighted a notable concentration of these voxels within the dmPFC subsystem, accounting for 71.5% of the significant voxels in the DMN. These findings suggest that the dmPFC subsystem’s role extends beyond mere semantic processing to include the integration of verbal information with various contextual elements. This association mechanism is particularly linked to verbal stimuli and their contexts. Supporting this specificity, a meta-analysis byKim (2011)demonstrated that associative versus item encoding tasks involving pictorial stimuli engage few regions within the dmPFC subsystem.

Alternative explanations for the heightened engagement of the dmPFC subsystem during associative word encoding warrant consideration. Firstly, word-word association tasks involve more complex semantic processing compared to encoding isolated words, which might suggest that the increased activity of the dmPFC subsystem is due to these elevated semantic demands. However, the detailed analysis of the individual studies within this meta-analysis revealed that the involvement of the dmPFC subsystem was not only confined to word-word associations but also extended to word-source tasks, where the increase in semantic demands was not as clearly evident as in word-word association tasks. Consequently, while increased semantic processing likely contributes to the enhanced engagement of the dmPFC subsystem in associative encoding tasks, it may not fully account for the observed phenomenon.

Secondly, associative encoding tasks are generally more demanding than item encoding tasks because they involve encoding both the item and its context. This increased complexity is evident in the current analysis of hit rates, which shows that associative encoding is more challenging. Although the corrected recognition rate did not significantly differ between the two encoding types, the difference in hit rates is noteworthy. This is because fMRI subsequent memory analyses specifically target old, studied trials, not new, unstudied trials. Thus, the enhanced engagement of the dmPFC subsystem in associative tasks might reflect its role in managing these more demanding encoding tasks. However, this interpretation contrasts with expectations that the frontoparietal network, known for supporting controlled or effortful processing (Dosenbach et al., 2007;Kim, 2019;Niendam et al., 2012;Zhang et al., 2017), would be predominantly engaged in such tasks. While the impact of task difficulty on encoding activity merits further exploration, categorizing tasks as merely difficult or easy may not adequately capture the nuances of the differential neural activity observed between associative and item word encoding.

Associative memory is commonly linked to recollection, while item memory tends to rely on both recollection and familiarity, as detailed byYonelinas (1997). This might suggest that the heightened activity in the dmPFC subsystem during associative tasks supports the creation of recollectable memories, rather than merely familiar ones. However, since recollection inherently requires integrating various elements of an experience into a cohesive whole, this observation does not introduce a new hypothesis but rather reinforces the existing understanding that the dmPFC subsystem is essential for encoding verbal information alongside its contextual elements, making a separate discussion redundant.

In summary, the enhanced engagement of the dmPFC subsystem in associative word encoding, compared to item word encoding, underscores its critical role in integrating verbal information with contextual details. However, tasks such as word-word associations involve significantly more semantic processing than encoding isolated words, and associative tasks in general pose greater challenges than item tasks. This complexity necessitates further research to disentangle how these factors specifically influence the activity of the dmPFC subsystem.

### 4.3 Comments on some null findings

Given the relatively small sample sizes in this meta-analysis, caution is warranted when interpreting null findings. This section addresses three notable instances. Firstly, prevailing theory suggests that the hippocampus supports associative encoding, while the perirhinal cortex is more engaged in item encoding (Davachi et al., 2003;Diana et al., 2007;Ranganath et al., 2004;Rugg et al., 2012). Contrary to these expectations, the comparison of item and associative encoding revealed no significant differences in MTL regions, even in the supplementary analysis with a lowered threshold. This suggests that the effects might be too subtle to detect, possibly due to low statistical power, or they might be absent altogether. However, these null results, alongside pronounced differences observed in the dmPFC subsystem, imply a more distinct role for this subsystem in associative compared to item word encoding.

Secondly, the initial analysis showed that specific regions in the left inferior frontal gyrus and left angular gyrus were more closely associated with associative than with item word encoding. However, the supplementary analysis that excluded studies with item-forgotten trials found that this association held true only for the left inferior frontal gyrus, but not for the left angular gyrus. The inconclusive results for the left angular gyrus—whether indicating a genuine absence of effects or due to low statistical power—necessitate cautious interpretation. Therefore, until more conclusive research is available, greater emphasis should be placed on the distinct role of the left inferior frontal gyrus in associative versus item word encoding.

Finally, the distinct analyses of item and associative word encoding effects demonstrated that item word encoding significantly engaged specific regions in the left middle frontal gyrus and left middle temporal gyrus, while associative word encoding did not. Nevertheless, the direct comparison between the two types of encoding did not identify any regions that were significantly more active during item word encoding compared to associative word encoding. These null results imply that any unique contributions of these regions to item versus associative word encoding may be subtle rather than pronounced.

### 4.4 Conclusions

This meta-analysis of fMRI-based subsequent memory studies provides two significant insights. First, both item and associative word encoding significantly engage the dmPFC subsystem of the DMN, underscoring its pivotal role in the semantic processing of incoming verbal information. While previous research has linked increased DMN activity to encoding interference, often attributed to the core subsystem’s propensity for mind-wandering, this study highlights the supportive function of the dmPFC subsystem in encoding, challenging traditional views and emphasizing a more nuanced understanding of the DMN’s role in memory processes. Second, the dmPFC subsystem, particularly its left inferior frontal component, is more actively engaged in associative word encoding than in item word encoding. This finding suggests that the subsystem’s role extends beyond basic semantic processing to include integrating verbal information with contextual elements. Moving beyond the traditional hippocampus-centric view in associative encoding research, this study positions the dmPFC subsystem as crucial, advocating for a broader perspective on associative mechanisms. Overall, this research enriches our understanding of the neural bases of memory encoding by clarifying the specific contributions of the DMN-dmPFC subsystem.

## Supplementary Material

Supplementary Material

## Data Availability

The data supporting the study findings are openly available athttps://identifiers.org/neurovault.collection:16575

## Utilization of Generative AI and AI-Assisted Technologies in the Writing Process

While preparing this manuscript, the author used ChatGPT to improve language and readability. After using this tool, the author reviewed and edited the content as needed and takes full responsibility for the content of the publication.
