# Supplementary material for: A default mode network subsystem supports both item and associative word encoding: Insights from a meta-analysis
Source: Imaging Neurosci (Camb). 2024 Oct 22;2:imag-2-00321. doi: 10.1162/imag_a_00321 (PMC12290809; doi:10.1162/imag_a_00321)
Supplement: Supplementary Material [file imag_a_00321-supp.pdf]

Supplementary Material for

A Default Mode Network Subsystem Supports Both Item and Associative Word Encoding: Insights from a Meta-Analysis

Hongkeun Kim
Department of Rehabilitation Psychology, Daegu University

Contents

Table S1. Overview of the studies included in the meta-analysis of item subsequent memory effects, as presented in the main text.

Table S2. Overview of the studies included in the meta-analysis of associative subsequent memory effects, as presented in the main text.

Supplementary references: Complete list of the studies utilized in the meta-analyses presented in the main text.

Table S1. Overview of the studies included in the meta-analysis of subsequent memory effects in item word encoding, as presented in the main text.

First author	Year	Subjects	Encoding stimulus category	Encoding task	Subsequent memory contrast
Axmacher	2008	30	Word	Working memory task involving delayed-match-to-sample	Correct working memory response trials: hit > miss
				Working memory task involving delayed-match-to-sample	Incorrect working memory response trials: hit > miss
Baker	2001	18	Word	Abstract/concrete judgment	hit > miss
				Uppercase/lowercase judgment	hit > miss
Bastin	2012	17	Word	Directed remembering/forgetting	hit > miss
Chee	2003	16	Word	Animacy judgment	hit > miss
Chee	2004	16	Word	Animacy judgment	hit > miss
Chen	2013	16	Word	Animacy judgment	hit > miss
de Zubicaray	2005	14	Word	Passive viewing	hit > miss
Evans	2017	40	Word	Determining if the word denotes a profession	hit > miss
Evans	2020	32	Word	Determining if the word denotes a profession	hit > miss
Fletcher	2003	9	Word	Pleasantness judgment/deciding whether the two underlined letters in the word are in alphabetical order	hit > miss
Henson	2005	22	Word	Deciding whether the first and last letters of the word are in alphabetical order	hit > miss
Honey	2005	12	Word	Pleasantness or syllable number judgment	hit > miss
Kircher	2008	29	Word	Intentional encoding	hit > miss

Reber	2002	12	Word	Directed remembering/forgetting	hit > miss
Weisenbach	2014	23	Word	Silent reading	hit > miss
Wimber	2010	20	Word	Syllable number judgment	hit > miss
Yang	2015	26	Word	Abstract/concrete judgment	hit > miss
Zierhut	2010	13	Word	Pleasantness or syllable number judgment	hit > miss

Table S2. Overview of the studies included in the meta-analysis of subsequent memory effects in associative word encoding, as presented in the main text.

First author	Year	Subjects	Encoding stimulus category	Encoding task	Association type	Subsequent memory contrast†
Addante	2015	20	Word–word pair	Deciding which of the 2 objects, denoted by the 2 words, would fit inside of the other	Word–word	Intact > rearranged
de Chastelaine	2014	18	Word–word pair	Relatedness judgment	Word–word	Intact > rearranged
de Chastelaine	2016	136	Word–word pair	Deciding which of the 2 objects, denoted by the 2 words, is more likely to fit into the other	Word–word	Intact > rearranged
Diana	2017	24	Word	Performing one of the four word categorization tasks: noun/verb, good/bad, nice/weird, common/uncommon	Word–task	Source-hit > source-miss
Gold	2006	15	Word	Imagining an indoor or outdoor scene associated with the word	Word–task	Source-hit > source-miss
Jackson	2004	12	Word–word pair	Forming a mental image incorporating the concepts represented by the 2 words	Word–word	Intact > rearranged
Maillet	2014	21	Word	Man-made/natural or pleasantness judgment	Word–task	Source-hit > all other responses
Park	2013	24	Word	Forming a mental image of the referent of the word in the background color and making a pleasantness judgment for the word/color combination	Word–color	Source-hit > source-miss
Park	2008a	20	Word–word pair	Semantic or phonological similarity judgment	Word–word	Intact > all other responses

Park	2008b	17	Word	Animacy judgment	Word–location	Source-hit > source-miss
				Syllable number judgment	Word–location	Source-hit > source-miss
Ranganath	2004	13	Word	Size or animacy judgment	Word–color	Source-hit > source-miss
Uncapher	2006	20	Word	Animacy judgment	Word–color–location	Both sources-hit> both sources-miss
				Animacy judgment	Word–location	Location only-hit > both sources-miss
				Animacy judgment	Word–color	Color only-hit > both sources-miss

Note. † The term 'intact' refers to trials where intact pairs were correctly recognized as being in their original state, while the term 'rearranged' denotes trials where intact pairs were mistakenly judged to be rearranged. The term 'source-hit' refers to trials where both the item and its associated source (context) were remembered correctly, while the term 'source-miss' refers to trials where the item was remembered correctly, but the source was remembered incorrectly

Supplementary references: Complete list of the studies utilized in the meta-analyses presented in the main text.

Addante, R.J., de Chastelaine, M., Rugg, M.D., 2015. Pre-stimulus neural activity predicts successful encoding of inter-item associations. NeuroImage. 105**,** 21-31.

Axmacher, N., Schmitz, D.P., Weinreich, I., Elger, C.E., Fell, J., 2008. Interaction of working memory and long-term memory in the medial temporal lobe. Cerebral Cortex. 18**,** 2868-2878.

Baker, J.T., Sanders, A.L., Maccotta, L., Buckner, R.L., 2001. Neural correlates of verbal memory encoding during semantic and structural processing tasks. NeuroReport. 12**,** 1251-1256.

Bastin, C., Feyers, D., Majerus, S., Balteau, E., Degueldre, C., Luxen, A., Maquet, P., Salmon, E., Collette, F., 2012. The neural substrates of memory suppression: a fMRI exploration of directed forgetting. PLoS One. 7**,** e29905.

Chee, M.W.L., Goh, J.O.S., Lim, Y., Graham, S., Lee, K., 2004. Recognition memory for studied words is determined by cortical activation differences at encoding but not during retrieval. NeuroImage. 22**,** 1456-1465.

Chee, M.W.L., Westphal, C., Goh, J., Graham, S., Song, A.W., 2003. Word frequency and subsequent memory effects studied using event-related fMRI. NeuroImage. 20**,** 1042-1051.

Chen, T.-C., Kuo, W.-J., Chiang, M.-C., Tseng, Y.-J., Lin, Y.-Y., 2013. Over-activation in bilateral superior temporal gyrus correlated with subsequent forgetting effect of Chinese words. Brain and Language. 126**,** 203-207.

de Chastelaine, M., Mattson, J.T., Wang, T.H., Donley, B.E., Rugg, M.D., 2016. The relationships between age, associative memory performance, and the neural correlates of successful associative memory encoding. Neurobiology of Aging. 42**,** 163-176.

de Chastelaine, M., Rugg, M.D., 2014. The relationship between task-related and subsequent memory effects. Human Brain Mapping. 35, 3687-3700.

de Zubicaray, G.I., McMahon, K.L., Eastburn, M.M., Finnigan, S., Humphreys, M.S., 2005. fMRI evidence of word frequency and strength effects in recognition memory. Cognitive Brain Research. 24, 587-598.

Diana, R.A., 2017. Parahippocampal cortex processes the nonspatial context of an event. Cerebral Cortex. 27, 1808-1816.

Evans, S.L., Dowell, N.G., Prowse, F., Tabet, N., King, S.L., Rusted, J.M., 2020. Mid age APOE ε4 carriers show memory-related functional differences and disrupted structure-function relationships in hippocampal regions. Scientific Reports. 10, 3110.

Evans, S., Dowell, N.G., Tabet, N., King, S.L., Hutton, S.B., Rusted, J.M., 2017. Disrupted neural activity patterns to novelty and effort in young adult APOE-e4 carriers performing a subsequent memory task. Brain and Behavior. 7, e00612.

Fletcher, P.C., Stephenson, C.M.E., Carpenter, T.A., Donovan, T., Bullmore, E.T., 2003. Regional brain activations predicting subsequent memory success: an event-related fMRI study of the influence of encoding tasks. Cortex. 39, 1009-1026.

Gold, J.J., Smith, C.N., Bayley, P.J., Shrager, Y., Brewer, J.B., Stark, C.E.L., Hopkins, R.O., Squire, L.R., 2006. Item memory, source memory, and the medial temporal lobe: concordant findings from fMRI and memory-impaired patients. Proceedings of the National Academy of Sciences of the United States of America. 103, 9351-9356.

Henson, R.N.A., Hornberger, M., Rugg, M.D., 2005. Further dissociating the processes involved in recognition memory: an fMRI study. Journal of Cognitive Neuroscience. 17, 1058-1073.

Honey, G.D., Honey, R.A.E., O'Loughlin, C., Sharar, S.R., Kumaran, D., Suckling, J., Menon, D.K., Sleator, C., Bullmore, E.T., Fletcher, P.C., 2005. Ketamine disrupts frontal and hippocampal contribution to encoding and retrieval of episodic memory: an fMRI study. Cerebral Cortex. 15, 749-759.

Jackson, O., Schacter, D.L., 2004. Encoding activity in anterior medial temporal lobe supports subsequent associative recognition. NeuroImage. 21, 456-462.

Kircher, T., Weis, S., Leube, D., Freymann, K., Erb, M., Jessen, F., Grodd, W., Heun, R., Krach, S., 2008. Anterior hippocampus orchestrates successful encoding and retrieval of non-relational memory: an event-related fMRI study. European Archives of Psychiatry and Clinical Neuroscience. 258**,** 363-372.

Maillet, D., Rajah, M.N., 2014. Dissociable roles of default-mode regions during episodic encoding. NeuroImage. 89**,** 244-255.

Park, H., Leal, F., Spann, C., Abellanoza, C., 2013. The effect of object processing in content-dependent source memory. BMC Neuroscience. 14**,** 71.

Park, H., Rugg, M., 2008a. Neural correlates of successful encoding of semantically and phonologically mediated inter-item associations. NeuroImage. 43**,** 165-172.

Park, H., Uncapher, M.R., Rugg, M.D., 2008b. Effects of study task on the neural correlates of source encoding. Learning and Memory. 15**,** 417-425.

Ranganath, C., Yonelinas, A.P., Cohen, M.X., Dy, C.J., Tom, S.M., D'Esposito, M., 2004. Dissociable correlates of recollection and familiarity within the medial temporal lobes. Neuropsychologia. 42**,** 2-13.

Reber, P.J., Siwiec, R.M., Gitleman, D.R., Parrish, T.B., Mesulam, M.-M., Paller, K.A., 2002. Neural correlates of successful encoding identified using functional magnetic resonance imaging. Journal of Neuroscience. 22**,** 9541-9548.

Uncapher, M.R., Otten, L.J., Rugg, M.D., 2006. Episodic encoding is more than the sum of its parts: an fMRI investigation of multifeatural contextual encoding. Neuron. 52**,** 547-556.

Weisenbach, S.L., Kassel, M.T., Rao, J., Weldon, A.L., Avery, E.T., Briceno, E.M., Ajilore, O., Mann, M., Kales, H.C., Welsh, R.C., 2014. Differential prefrontal and subcortical circuitry engagement during encoding of semantically related words in patients with late-life depression. International Journal of Geriatric Psychiatry. 29**,** 1104-1115.

Wimber, M., Heinze, H.-J., Richardson-Klavehn, A., 2010. Distinct frontoparietal networks set the stage for later perceptual identification priming and episodic recognition memory. Journal of Neuroscience. 30**,** 13272-13280.

Yang, H., Cai, Y., Liu, Q., Wang, Q., Zhao, X., Chen, C., Xue, G., 2015. Differential neural correlates underlie judgment of learning and subsequent memory performance. Frontiers in Psychology. 6, 1699.

Zierhut, K., Bogerts, B., Schott, B., Fenker, D., Walter, M., Albrecht, D., . . . Schiltz, K., 2010. The role of hippocampus dysfunction in deficient memory encoding and positive symptoms in schizophrenia. Psychiatry Research: Neuroimaging. 183, 187-194.
